# P-1618. A Retrospective Analysis of Co- and Superinfections with COVID-19 in the Post-Omicron Era

**DOI:** 10.1093/ofid/ofaf695.1795

**Published:** 2026-01-11

**Authors:** Hasna S Karim, Courtney E Harris, Drew W Charles, Alexandra G Mills, Zachary P Gruss, Richard R Lueking, Adam G Fine

**Affiliations:** Medical University of South Carolina, Charleston, SC; Medical University of South Carolina, Charleston, SC; Medical University of South Carolina, Charleston, SC; Medical University of South Carolina, Charleston, SC; Medical University of South Carolina, Charleston, SC; Medical University of South Carolina, Charleston, SC; University of Chicago, Chicago, Illinois

## Abstract

**Background:**

Upon COVID-19’s emergence, clinicians faced uncertainty in managing the new pathogen and associated secondary infections that increased its morbidity and mortality. Analyses from 2020 to 2022 characterized the epidemiology of such infections, but data beyond 2023 are scarce, representing a gap in data reflective of the current state of the pandemic.

**Figure:**
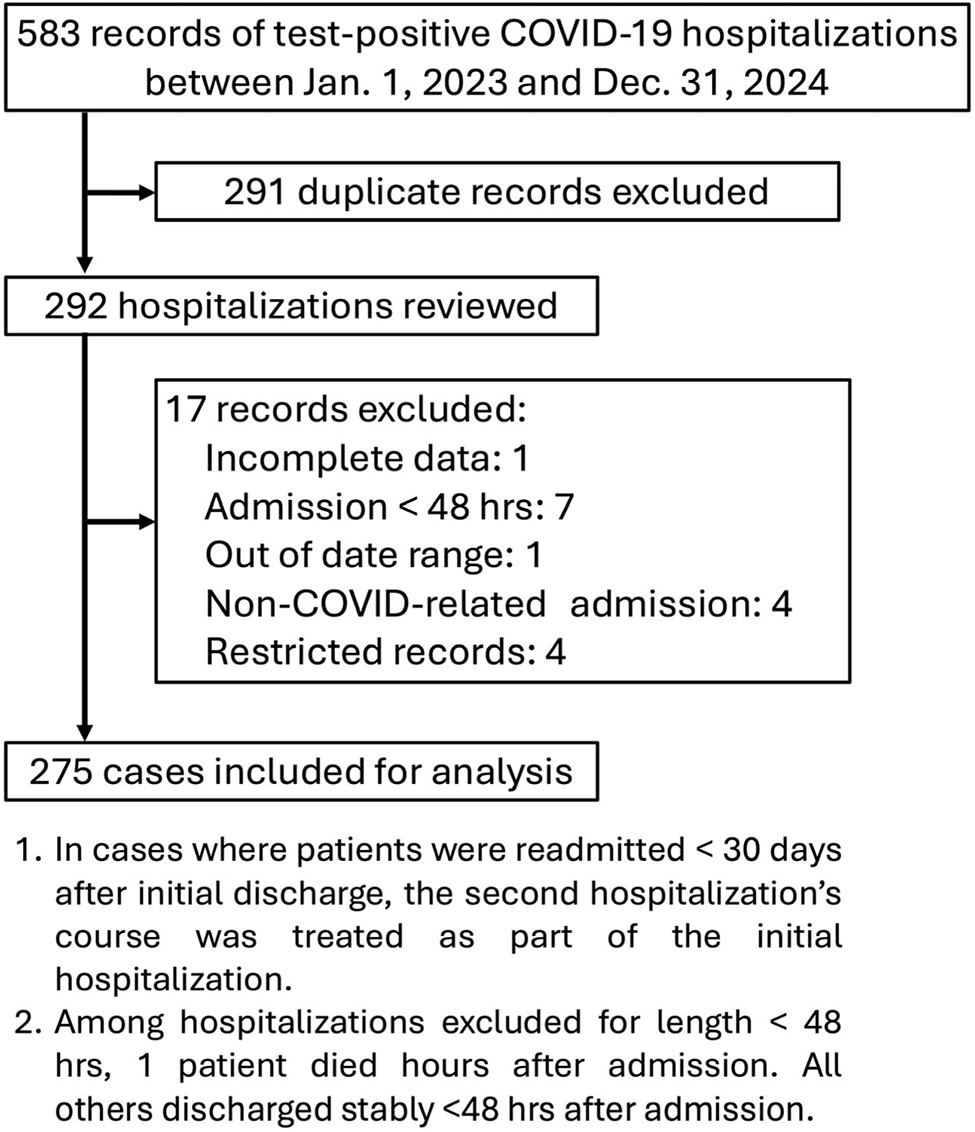
Study flowchart

**Figure:**
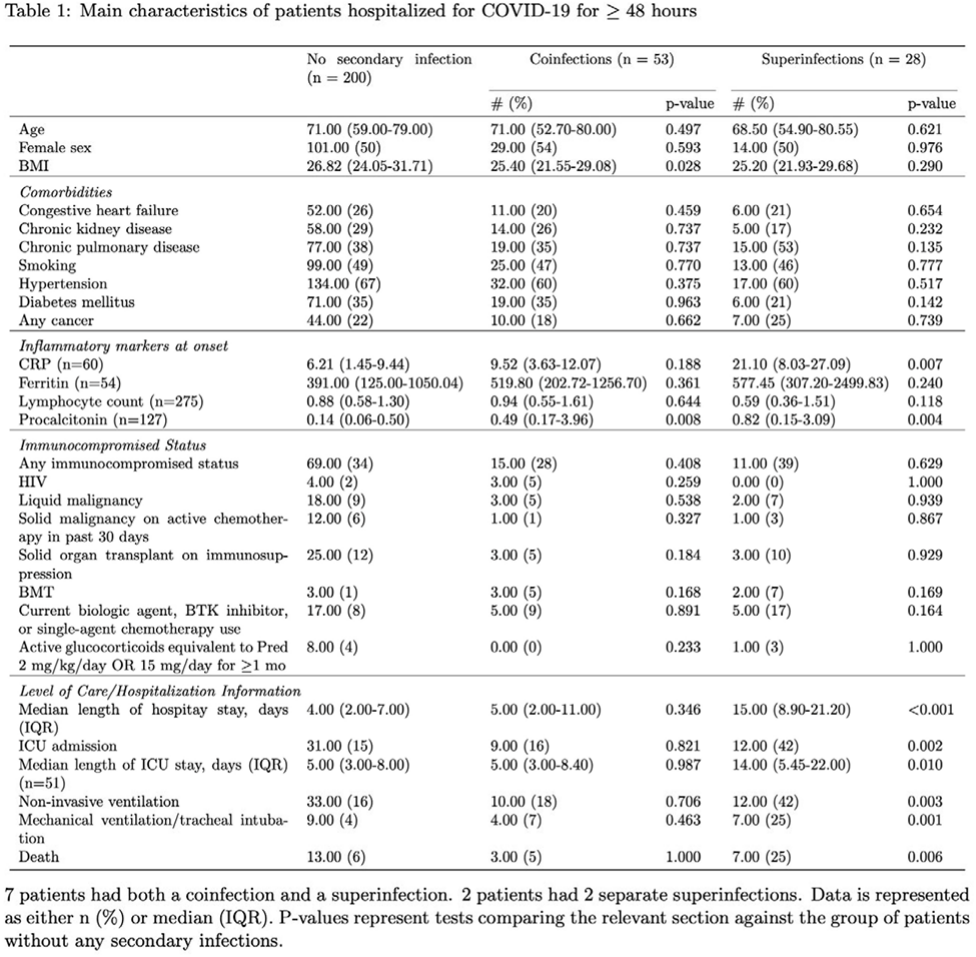

**Methods:**

A retrospective cohort analysis of adult COVID-19 hospitalizations at Medical University of South Carolina between Jan. 1, 2023 and Dec. 31, 2024 was performed via electronic medical record (see Figure 1 for exclusion criteria) to obtain demographics and microbiological results. The primary outcome was the presence of secondary infection defined as either coinfection (diagnosis < 48 hours after admission) or superinfection (diagnosis > 48 hours after admission) by either clinical or microbiological criteria.

**Figure:**
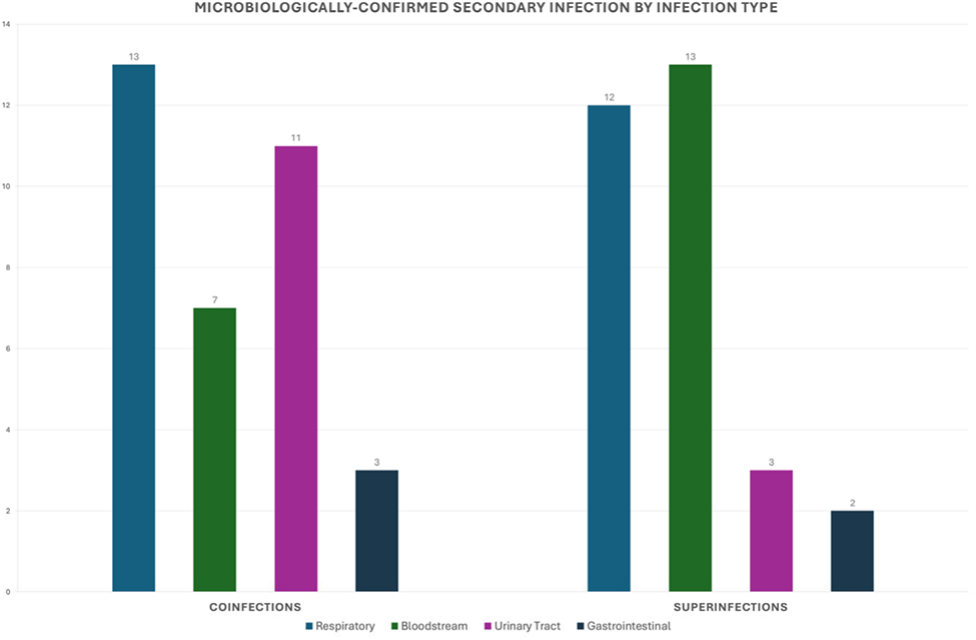
Breakdown of secondary infections by affected organ system Seven patients had both co- and superinfections; thirteen patients had multiple sites of infection.

**Figure:**
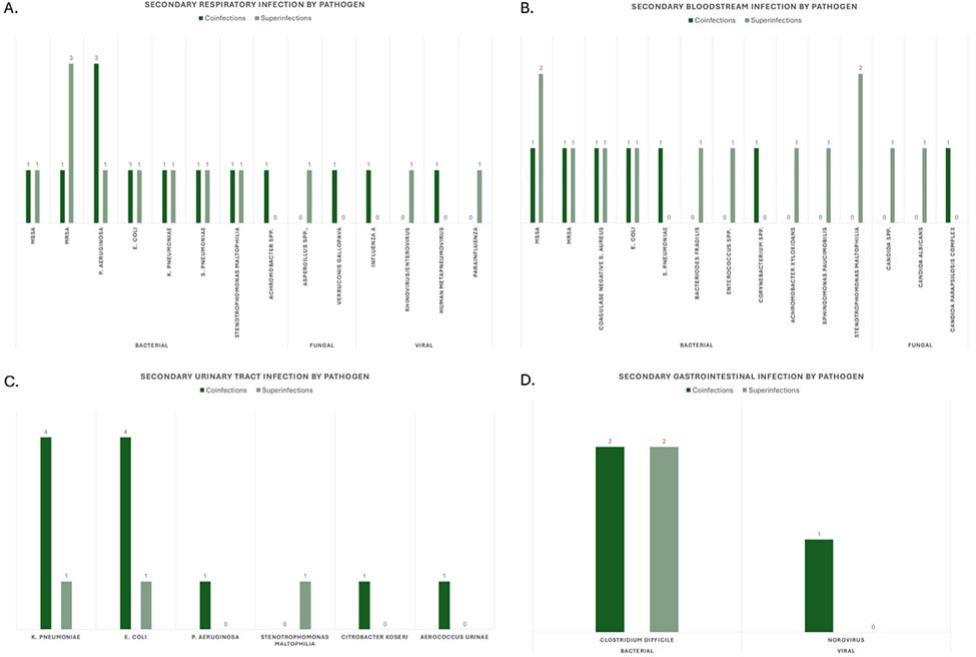
Secondary infection by pathogen Patients could have multiple sites of infection and multiple associated pathogens.

**Results:**

Of 275 adults hospitalized with COVID-19, 75 patients were diagnosed with any secondary infection (27%), and of these 7 (9%) had both co- and superinfection and 13 (17%) had infections of multiple organ systems (Table 1, Figure 2). Pathogens causing secondary infections are outlined in Figure 3. Fifty three patients (19%) had a coinfection, and of these 25 (47%) met clinical and microbiological criteria for infection (79% bacterial) while 28 (53%) met clinical criteria only. Twenty eight patients (10%) had a superinfection, and of these 21 (75%) met clinical and microbiological criteria for infection (57% bacterial) while 7 (25%) met clinical criteria only. Superinfections were associated with longer hospital (p < 0.001) and ICU stays (p = 0.001), need for non-invasive (p = 0.003) and mechanical ventilation (p = 0.001), and higher mortality (p = 0.006). No subset of immunocompromised status was significantly associated with greater risk of secondary infection.

**Conclusion:**

Our analysis shows an increase in aggregate secondary infection incidence from early-pandemic studies. Rates of microbiologically-confirmed coinfections and superinfections remain low; however, rates of clinically-diagnosed secondary infections may be higher than previously reported. Superinfections remain associated with greater morbidity and mortality in our cohort, while coinfections were not associated with worse outcomes.

**Disclosures:**

All Authors: No reported disclosures

